# Multinational investigation of a *Salmonella* Umbilo outbreak reveals rocket salad and baby spinach as the likely infection vehicles, Europe, 2024

**DOI:** 10.2807/1560-7917.ES.2024.29.46.2400728

**Published:** 2024-11-14

**Authors:** Bettina M Rosner, Sandra Simon, Stine Nielsen, Sandra Köberl-Jelovcan, Pernille Gymoese, Dirk Werber, Anika Meinen, Michael Pietsch, Antje Flieger, Jennie Fischer, Marina C Lamparter, Felix Küffel, Fiona Költringer, Christian Kornschober, Luise Müller, Gerhard Falkenhorst, Sabine Maritschnik

**Affiliations:** 1 Department of Infectious Disease Epidemiology; Robert Koch Institute, Berlin, Germany; 2 Unit of Enteropathogenic Bacteria and *Legionella* and National Reference Centre for *Salmonella* and Other Bacterial Enteric Pathogens, Department of Infectious Diseases, Robert Koch Institute, Wernigerode, Germany; 3 Department of Infectious Disease Epidemiology and Prevention; Statens Serum Institut, Copenhagen, Denmark; 4 National Reference Centre for *Salmonella*; Austrian Agency for Health and Food Safety, Graz, Austria; 5 Department of Bacteria, Parasites and Fungi; Statens Serum Institut, Copenhagen, Denmark; 6 Institute for Infectious Disease Epidemiology; Austrian Agency for Health and Food Safety, Vienna, Austria; 7 National Reference Laboratory for *Salmonella*, German Federal Institute for Risk Assessment (BfR), Berlin, Germany; * These authors contributed equally to this work and share first authorship.; ** These authors contributed equally to this work and share last authorship.

**Keywords:** food-borne, infections, *Salmonella*, outbreaks, *S.* Umbilo, rocket salad, spinach

## Abstract

A food-borne outbreak with about 200 *Salmonella* Umbilo cases occurred mainly between July and September 2024 in several European countries. Collaborative work between outbreak teams in Germany, Austria and Denmark, including epidemiological and microbiological investigations, allowed to rapidly identify rocket salad as the likely infection vehicle. *Salmonella* Umbilo was detected in rocket salad, and later in baby spinach. The food isolates and clinical outbreak strain were genetically closely related. Both food items originated from the same company in Italy.

We report on the collaborative investigation of an outbreak including about 200 cases of *Salmonella enterica* serotype Umbilo (*S*. Umbilo) that affected several European countries mainly between July and September 2024. The investigation enabled to rapidly identify and microbiologically confirm rocket salad (also known as arugula or rucola) from company A in Italy as the likely food vehicle. Baby spinach and possibly other items produced by the same company could be additional food vehicles. While case numbers appeared to decline in October 2024, some *S*. Umbilo infections (with genomic analysis pending to confirm these cases) continue to be detected at the time of writing.

## Outbreak detection and early epidemiological investigations

On 14 August 2024 the Robert Koch Institute (RKI), the national public health institute in Germany, observed an unusual increase of clinical *S*. Umbilo isolates (antigenic formula 28:z10:e,n,x [1]) and corresponding case notifications. At the time, the national reference centre for *Salmonella* and other bacterial enteric pathogens (NRC) had registered nine *S*. Umbilo isolates since July, compared to a median of three isolates annually in the previous 5 years. Core-genome multilocus sequence typing (cgMLST; EnteroBase scheme in RidomSeqSphere^+^) revealed that these isolates were genetically closely related (0–3 allelic differences (AD)). Subsequently, numbers of notified cases in Germany increased. In weeks 31–35 (29 July–1 September) 16–21 cases fell ill per week. The temporal and spatial distribution of cases all over Germany suggested that the outbreak was probably caused by contaminated food item(s) that had been available nationwide since July 2024.

Most cases occurred in weeks 31 to 35, 2024 (end of July to end of August 2024). Due to the inevitable delay between disease onset and case identification, additional cases with later disease onsets may still be detected.

On 19 August 2024 Germany alerted other countries about the unusual increase of *S*. Umbilo cases via EpiPulse, the European surveillance portal for infectious diseases of the European Centre for Disease Prevention and Control (ECDC), and, on 27 September 2024, via the Early Warning Response System (EWRS) of the European Commission. Denmark and Austria were the first countries to also report *S*. Umbilo cases in response to Germany’s EpiPulse alert. Up to 18 October 2024, Denmark and Austria had identified 22 and 19 *S*. Umbilo outbreak cases, respectively, and six other European countries reported between one and 20 *S*. Umbilo cases each via EpiPulse.

Exploratory case interviews were conducted by the RKI in Germany using a standard questionnaire that was adapted to this outbreak. Interview results suggested an association of *S*. Umbilo infections with consumption of rocket salad and/or other leafy green salads in the 3 days before disease onset. This information was also shared via EpiPulse.

## Collaborative outbreak investigation in Germany, Denmark and Austria

Public health institutes in Germany, Denmark, and Austria collaborated closely in the outbreak investigation by rapidly exchanging information about results from case interviews and other investigations. A common case definition was applied. A confirmed case was defined as an individual with disease onset or, alternatively, notification date or laboratory (testing or registration) date, on or after 1 July 2024 and whose corresponding *S*. Umbilo isolate belonged to the outbreak cluster with ≤ 5 AD to the reference outbreak strain by cgMLST. A probable case was defined as an individual with disease onset date (or notification or laboratory date) on or after 1 July 2024 who was notified in the routine surveillance system as an *S*. Umbilo case without a corresponding isolate, or with a corresponding isolate that had not (yet) been sequenced.

**Figure 1:**
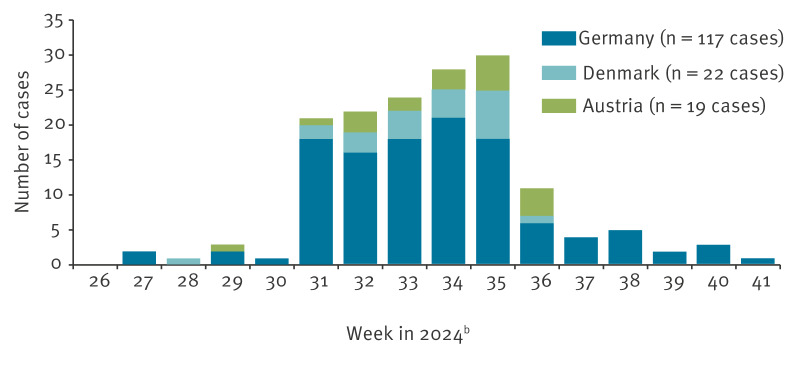
*Salmonella* Umbilo outbreak cases by calendar week of disease onset, Germany, Denmark and Austria, 24 June−13 October 2024 (n = 158 cases)^a^

^a^ The graph is based on the available data up to 18 October 2024. One case lacking information regarding the week of disease onset and laboratory data could not be included.

^b^ Cases are shown by week of disease onset when known, or by estimated week of disease onset, when the date of disease onset was unknown. The estimated week of disease onset was determined as the week of notification or laboratory (testing or registration) date minus 1 week.

Up to 18 October 2024, 159 cases were reported in the three countries alone (118 in Germany, 22 in Denmark, 19 in Austria). Of those, 149 were confirmed cases. The first reported date of disease onset was 1 July 2024, the latest was 4 October 2024 (Figure 1). The median age of cases was 37 years (range: 1–91 years; interquartile range: 26–56 years). Male and female individuals were equally affected (80 female, 79 male). Of 129 cases with complete information on hospitalisation, 36 (28%) were hospitalised because of salmonellosis for at least 12 hours (DK) or at least one night (DE, AT). Up to 18 October 2024, one person (a confirmed case) was reported as deceased due to salmonellosis.

In Germany, 42 cases were interviewed. Of those, 37 reported consuming leafy green salads in the 3 days before disease onset, and 24/42 specifically reported consumption of rocket salad (Table).

**Table:** Results of exploratory *Salmonella* Umbilo case interviews in Germany, Denmark and Austria regarding the consumption (or likely consumption) of rocket salad and/or another leafy green salad in the days^a^ before disease onset, 2024 (n = 159 cases)

Country	Number of reported cases	Number of interviewed cases	Proportion of cases who ate rocket salad	%	Proportion of cases who ate rocket salad or another type of leafy green salad	%
DE	118	42	24/42	NA	37/42	NA
DK	22	14	8/14	NA	12/14	NA
AT	19	18	12/18	NA	18/18	NA
**Overall**	**159**	**74**	**44/74**	**59**	**67/74**	**91**

AT: Austria; DE: Germany; DK: Denmark; NA: not applicable, as percentages based on small numbers would not be meaningful.

^a^ In DE, food history was queried for the 3 days before disease onset, in AT and DK, for the 7 days before disease onset.

In Austria, all 18 interviewed cases reported consumption of leafy green salads in the 7 days before onset of illness, and 12/18 cases specifically mentioned eating rocket salad (Table). In addition, case interviews identified several cafés and food business operators where patients had consumed rocket salad. Backward tracing along the food supply chain of rocket salad served in café X on 15 August 2024 revealed that the salad had been distributed by company A and originated from production region A in Italy.

In Denmark, 12/14 interviewed patients reported eating different types of leafy green salads. Some of them (8/14) reported consumption of rocket salad and 10/14 reported consumption of either rocket salad or baby spinach in the 7 days before their illness began. Investigations that involved consumer purchase data showed that several Danish cases had likely bought rocket salad or baby spinach distributed by company A in the days before disease onset.

## Microbiological investigations of clinical and food isolates

**Figure 2:**
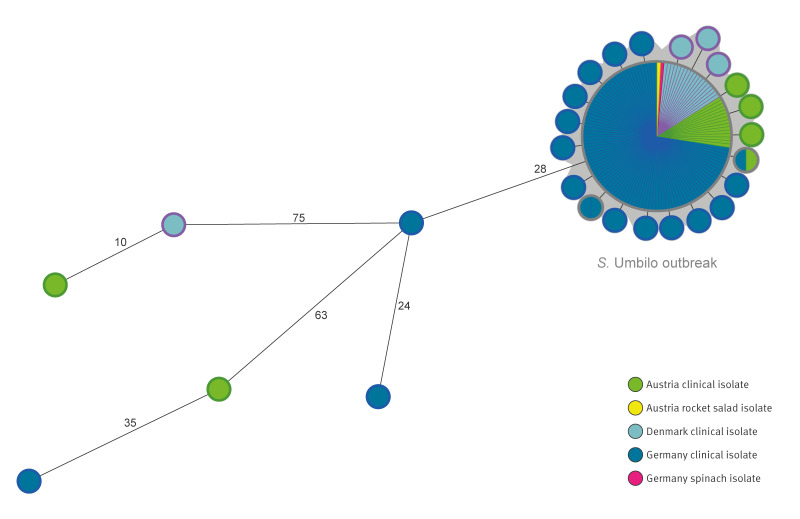
Minimum spanning tree based on genetic sequences of clinical (n = 155) and food (n = 3) *Salmonella* Umbilo outbreak isolates, Germany, Austria and Denmark, 2024 (n = 158 isolates)

Clinical isolates from Germany, Austria, and Denmark were genetically closely related with a maximum pairwise distance of 4 AD and a maximum distance of 2 AD to the reference sequence, respectively (Figure 2). *S*. Umbilo isolates that differed by > 5 AD from the clinical outbreak reference strain were not considered part of this outbreak. The clinical outbreak strain reference sequence (24–05800) was deposited in EnteroBase (ID RKI_24–05800; EnteroBase cgMLST HC5_93993) and the European Nucleotide Archive (ENA) under accession number ERR13934259 in BioProject PRJEB67705.

cgMLST: core-genome MLST; MLST: multilocus sequence typing; MST: minimum spanning tree

Ridom SeqShere^+^ MST for 158 samples based on 3,002 columns, pairwise ignoring missing values, logarithmic scale. Distance is based on columns from *S*. *enterica* cgMLST Enterobase (3,002), *S*. *enterica* MLST (7). MST cluster threshold is five allelic differences [14,15] and affiliation of isolates to the outbreak cluster is indicated by grey shading in the MST (149 clinical isolates, 3 food isolates). Allelic distances to the outbreak reference strain, which are higher than five, are indicated on the branches of the MST.

In Austria, nine samples of rocket salad were collected by food safety authorities from a café and food business operator mentioned during the case interviews. Two different rocket salad samples that were distributed by company A and originated from production region A in Italy tested positive for *S*. Umbilo (method according to EN ISO 6579–1:2017/A1:2020 [2]). On 20 September 2024, Austria shared this information via the European Rapid Alert System for Food and Feed (RASFF; notification 2024.7033). Subsequent sequencing confirmed that the rocket salad isolates belonged to the outbreak cluster (0 AD to the outbreak reference sequence), based on analysis of whole genome sequences (WGS). The sequence of the rocket salad isolate was deposited as National Center for Biotechnology Information (NCBI) BioSample ID SAMN44603168, BioProject PRJNA1182708.

In Germany, *Salmonella* was detected in a sample of organic baby spinach collected on 28 August 2024 at a supermarket as part of the routine zoonosis monitoring. For the detection of *Salmonella* from baby spinach the first enrichment step of method EN ISO 6579–1:2017/A1:2020 [2] was adjusted using buffered peptone water containing novobiocin (12 mg/L) as part of a *Salmonella* PCR pre-screening according to the ‘SureTect Salmonella Species PCR Assay’ (Thermo Fisher Scientific, Waltham, Massachussets, United States; catalogue number: A56841). The *Salmonella* isolate was later sent to the national reference laboratory (NRL) for *Salmonella* where it tested positive for *S*. Umbilo. This information was shared by the responsible food safety authorities via RASFF (notification 2024.7478; 10 October 2024). Subsequently, genome-based attribution of the *S*. Umbilo isolate from baby spinach to the outbreak WGS cluster was confirmed (0 AD to the rocket salad isolates and the outbreak reference strain). The sequence of the baby spinach isolate was deposited as NCBI BioSample ID SAMN44599062, BioProject PRJNA937468. The organic baby spinach had also been distributed by company A in Italy. Based on epidemiological investigations, the outbreak team in Denmark had already suspected baby spinach as a possible additional food vehicle. 

Sequences of 18 other *S*. Umbilo isolates (time period 2020–2024) from food, feed, animal, and environmental matrices were analysed by the NRL for *Salmonella* in Germany to investigate other possible sources, but none of them were genetically closely related to the outbreak reference strain (> 30 AD; based on ChewieSnake cgMLST workflow [3]).

## Outbreak control measures

After being alerted by Austria on 20 September 2024 via RASFF, Italian food safety authorities took actions to stop distribution of rocket salad and all products containing rocket salad from certain producers in region A in Italy, and ordered a recall of rocket salad from these producers from the market (RASFF 2024.7033). In Denmark, a press release was issued to inform the general public about the outbreak and to reinforce recommendations to always wash salads and vegetables before consumption. The Austrian response to the outbreak included swift action from wholesalers, who halted all distribution of rocket salad from company A and even switched suppliers entirely. By the time the positive laboratory results were confirmed, the batch in question was no longer available on the market, however consumers were informed about the health risks associated with the contaminated rocket salad. In Germany, the RKI informed about the outbreak in its Epidemiological Bulletin on 26 September 2024. The information was taken up by various media. 

## Discussion

Strong evidence from epidemiological, microbiological and product-tracing investigations generated by the outbreak investigation teams in Germany, Austria, and Denmark suggests that this international *S*. Umbilo outbreak was associated with rocket salad and possibly baby spinach from company A in Italy.

The *Salmonella* serotype Umbilo is usually rare, with 20 to 32 annual cases registered by the ECDC for the European Union/European Economic Area (including the United Kingdom (UK) until 2019) in the past 10 years (2014−2023) [4]. With about 200 cases among at least nine European countries, the current outbreak is, to our knowledge, the first reported with *S.* Umbilo in humans. In 2001 in the UK, *S*. Umbilo was detected in organic rocket salad and ready-to-eat bagged salads from a grower in Italy, but not linked to human cases [5].

Rocket salad has been described as the infection vehicle in several outbreaks caused by *Salmonella* or other bacteria or viruses. Examples are a *Salmonella* Typhimurium outbreak in Sweden in 2022 associated with Swedish rocket salad [6], a *Salmonella* Thompson outbreak in Norway in 2004, and *Salmonella* Napoli cases in Sweden in 2008−2009 linked to rocket salad imported from Italy [7,8], an outbreak in Finland in 2016 by Shigatoxin-producing and enteropathogenic *Escherichia coli* linked to rocket salad produced in Denmark [9], and a hepatitis-A virus outbreak in Sweden in 2000−2001 [10].

Food-borne outbreaks associated with fresh produce, including leafy green salads, are a challenging public health concern, because these food items are widely promoted as part of a healthy diet and are eaten uncooked [11]. Contamination of fresh produce may occur at various steps along the food-production chain, for example, when using animal manure as fertilizer, contaminated water for irrigation or for pre-washing of produce [12,13]. Contamination by animals on the production site, such as field lizards, may also play a role [5]. It remains to be elucidated how the rocket salad and baby spinach were contaminated in the *S*. Umbilo outbreak described here.

Genomic analyses have become an important tool for pathogen surveillance, and also provide information about strain characteristics, including the serotype. However, classical serotyping may be faster than sequencing methods, especially in countries with limited sequencing capacity and/or lack of automated bioinformatic workflows. In this outbreak, the rapid identification of the rare serotype *S*. Umbilo in clinical isolates by classical serotyping was crucial for its timely detection. Therefore, a suitable combination of phenotypic and genomic methods is beneficial for pathogen surveillance and outbreak detection when complete and timely genomic surveillance is not yet established.

The rapid identification of the suspected food vehicle in this outbreak was made possible by the close collaboration between the three countries that had the most cases early on (Germany, Denmark and Austria). Sharing results from the outbreak investigations in the different countries openly and swiftly via EpiPulse and other more direct communication pathways, such as emails, video conferences and telephone calls, was essential to the success of the investigations.

Our investigations have some limitations. In foodborne outbreaks the ascertainment of exposures often depends on patients’ recall of what they have eaten. In the present outbreak, most cases could only be interviewed approximately 2−4 weeks after onset of symptoms, which may have affected their recall ability. The main reason for the delay was the fact that serotyping (and thereby recognition as a probable outbreak case) was only done at the NRCs once they had received a patient’s *Salmonella* isolate from primary clinical laboratories. Furthermore, one might have eaten fresh produce like rocket salad and baby spinach unknowingly if it was part of a mixed salad or a composite food like a sandwich. The true size of the outbreak is unknown because not all persons with acute gastroenteritis seek medical care and microbiological diagnostics is not performed in all cases who do.

Italian food safety authorities ordered measures to stop the outbreak. Because of their short shelf life, rocket salad and baby spinach from the early lots with proven contamination have presumably been consumed or discarded by now. Since then, *S*. Umbilo and other *Salmonella* serotypes have been found in additional lots of rocket salad, including a lot originating from region A in Italy, which was sampled as recently as mid-October from a supermarket in Germany. Therefore, the situation requires further attention to ensure that no further cases of salmonellosis will occur due to contaminated rocket salad or other leafy green salads.

## Conclusions

This outbreak serves as a reminder that *Salmonella* can occur in fresh produce, including leafy green salads, intended for consumption without prior heating. Close collaboration between affected countries played a key role to timely identify the causative food vehicles. These may have been contaminated at one or more of different steps of their production and distribution. Therefore, identifying the ultimate source of contamination and controlling the outbreak can present challenges.

## References

[1] Lapage SP Taylor J Nicewonger CR Phillips AG . New serotypes of Salmonella identified before 1964 at the Salmonella reference laboratory, Colindale. Int J Syst Bacteriol. 1966;16(3):253-97. 10.1099/00207713-16-3-253

[2] Standard EN ISO 6579-1:2017/A1:2020: Microbiology of the food chain - Horizontal method for the detection, enumeration and serotyping of Salmonella - Part 1: Detection of Salmonella spp. 10.1016/j.ijfoodmicro.2018.03.022 29803313

[3] Deneke C Uelze L Brendebach H Tausch SH Malorny B . Decentralized investigation of bacterial outbreaks based on hashed cgMLST. Front Microbiol. 2021;12:649517. 10.3389/fmicb.2021.649517 34220740 PMC8244591

[4] European Centre for Disease Prevention and Control (ECDC). ECDC Surveillance Atlas of Infectious Diseases. Stockholm: ECDC. [Accessed 15 Oct 2024]. Available from: https://www.ecdc.europa.eu/en/surveillance-atlas-infectious-diseases ha

[5] Sagoo SK Little CL Ward L Gillespie IA Mitchell RT . Microbiological study of ready-to-eat salad vegetables from retail establishments uncovers a national outbreak of salmonellosis. J Food Prot. 2003;66(3):403-9. 10.4315/0362-028X-66.3.403 12636292

[6] Fischerström K Dryselius R Lindblad M Kühlmann-Berenzon S Karamehmedovic N Börjesson S Outbreak of *Salmonella* Typhimurium linked to Swedish pre-washed rocket salad, Sweden, September to November 2022. Euro Surveill. 2024;29(10):2300299. 10.2807/1560-7917.ES.2024.29.10.2300299 38456218 PMC10986667

[7] Nygård K Lassen J Vold L Andersson Y Fisher I Löfdahl S Outbreak of Salmonella Thompson infections linked to imported rucola lettuce. Foodborne Pathog Dis. 2008;5(2):165-73. 10.1089/fpd.2007.0053 18361685

[8] Graziani C Busani L Dionisi AM Caprioli A Ivarsson S Hedenström I Virulotyping of Salmonella enterica serovar Napoli strains isolated in Italy from human and nonhuman sources. Foodborne Pathog Dis. 2011;8(9):997-1003. 10.1089/fpd.2010.0833 21561382

[9] Kinnula S Hemminki K Kotilainen H Ruotsalainen E Tarkka E Salmenlinna S Outbreak of multiple strains of non-O157 Shiga toxin-producing and enteropathogenic *Escherichia coli* associated with rocket salad, Finland, autumn 2016. Euro Surveill. 2018;23(35):1700666. 10.2807/1560-7917.ES.2018.23.35.1700666 30180926 PMC6124187

[10] Nygård K Andersson Y Lindkvist P Ancker C Asteberg I Dannetun E Imported rocket salad partly responsible for increased incidence of hepatitis A cases in Sweden, 2000-2001. Euro Surveill. 2001;6(10):151-3. 10.2807/esm.06.10.00380-en 11891384

[11] Yang X Scharff R . Foodborne illnesses from leafy greens in the United States: attribution, burden, and cost. J Food Prot. 2024;87(6):100275. 10.1016/j.jfp.2024.100275 38609013

[12] Machado-Moreira B Richards K Brennan F Abram F Burgess CM . Microbial Contamination of Fresh Produce: What, Where, and How? Compr Rev Food Sci Food Saf. 2019;18(6):1727-50. 10.1111/1541-4337.12487 33336968

[13] Carstens CK Salazar JK Darkoh C . Multistate outbreaks of foodborne illness in the United States associated with fresh produce from 2010 to 2017. Front Microbiol. 2019;10:2667. 10.3389/fmicb.2019.02667 31824454 PMC6883221

[14] Zhou Z Alikhan NF Mohamed K Fan Y Achtman M Agama Study Group . The EnteroBase user’s guide, with case studies on *Salmonella* transmissions, *Yersinia pestis* phylogeny, and *Escherichia* core genomic diversity. Genome Res. 2020;30(1):138-52. 10.1101/gr.251678.119 31809257 PMC6961584

[15] Jünemann S Sedlazeck FJ Prior K Albersmeier A John U Kalinowski J Updating benchtop sequencing performance comparison. Nat Biotechnol. 2013;31(4):294-6. 10.1038/nbt.2522 23563421

